# Mutual relations between jasmonic acid and acibenzolar-*S*-methyl in the induction of resistance to the two-spotted spider mite (*Tetranychus urticae*) in apple trees

**DOI:** 10.1007/s10493-020-00539-6

**Published:** 2020-08-28

**Authors:** Wojciech Warabieda, M. Markiewicz, D. Wójcik

**Affiliations:** Research Institute of Horticulture, 1/3 Konstytucji 3 Maja, 96-100 Skierniewice, Poland

**Keywords:** Mites, Induced resistance, Salicylic acid, Benzothiadiazole, Antagonism

## Abstract

The possibility of inducing resistance to the two-spotted spider mite, *Tetranychus urticae* Koch, in ‘Gala’ apple trees growing under optimal fertilization or nitrogen-deficiency conditions was investigated. The effects of jasmonic acid (JA) at 1.5 and 2.5 mM, and acibenzolar-*S*-methyl (benzothiadiazole, BTH) at 0.5 and 1.5 mM, applied separately or together, on the fecundity of *T. urticae* females in a laboratory test as well as on the population growth of the pest in a greenhouse experiment were determined. The influence of both elicitors on the induction of LOX and PAL gene expression was assessed in a parallel experiment using real-time PCR. Jasmonic acid showed significantly higher effectiveness in inducing apple tree resistance to *T. urticae*, as compared to BTH. This was particularly evident in the reduction in pest numbers that was observed in the greenhouse experiment and was also confirmed by increased LOX gene expression after treatment with JA. BTH induced the expression of the PAL gene more strongly than jasmonic acid; however, this was not reflected in the performance of the two-spotted spider mite in the laboratory and greenhouse experiments. It was also found that the antagonistic effect of BTH on JA might lead to decreased effectiveness of the jasmonic acid used to induce apple tree resistance to the two-spotted spider mite. Although nitrogen fertilization stimulated the development of spider mite populations, the resistance induction mechanism was more effective in N-fertilized plants, which was especially evident at the higher jasmonic acid concentration.

## Introduction

Spider mites, including the two-spotted spider mite, *Tetranychus urticae* Koch, are harmful pests of apple orchards, which can build up very large populations causing severe economic losses. Such a situation often occurs in orchards with an imbalance between pests and predators as a result of incorrect management using non-selective pesticides. For many years, there has been a tendency in agriculture to reduce the use of pesticides. It relates to the increasing public awareness concerning the negative effects of excessive use of chemicals in agriculture on the environment and human health. With the increased interest in limiting the use of pesticides, the integrated pest management (IPM) method of suppressing populations of pathogens or pests has been developed. Due to the limitation in the number of active substances allowed for use in plant protection, alternative methods of controlling agrophages have been sought in recent years. One of them relies on inducing the natural defence mechanisms in plants.

The induction of plant resistance mechanisms to many stresses, including pathogens and pests, is a very complicated process. It is generally considered that salicylic acid (SA) and jasmonic acid (JA) with the participation of ethylene (E) are the main signal molecules that regulate the expression of many defence genes (Bari and Jones 2009; Gimenez-Ibanez and Solano 2013). Salicylic acid is regarded as a signal molecule in plant defence against biotroph pathogens (Bari and Jones 2009; Smith et al. 2009) and can thus be used in plant protection practice. For example, acibenzolar-*S*-methyl (benzothiadiazole, BTH), a functional analogue of SA, has been commercialized and proved to be useful in the protection against fire blight of apple and pear trees (Maxson-Stein et al. 2002; Norelli et al. 2003; Sparla et al. 2004; Bazzi et al. 2006).

The first enzyme in the SA biosynthesis pathway is phenylalanine ammonia lyase (PAL). *PAL* is known to be induced by many stresses such as salinity stress, low temperatures, tissue wounding and pathogen attack (Campos-Vargas and Saltveit 2002; Dehghan et al. 2014; González-Candelas et al. 2010; Nyaka Ngobisa et al. 2016; Pääkkönen et al. 1998; Sayari et al. 2014; Valifard et al. 2015). Phenylalanine ammonia lyase converts l-phenylalanine to ammonia and trans-cinnamic acid, leading to biosynthesis of phenylpropanoid natural products such as lignin, pigments, flavonoids, and phytoalexins, via phenylpropanoid and flavonoid pathways (Solecka and Kacperska 2003). The PAL enzyme seems to have an important role in plant defence against herbivorous insects and mites. Increased expression of *PAL* was detected in tomato as a result of spider mite feeding (Ament et al. 2004). Similarly, an increased level of PAL gene expression was observed during the feeding of aphids on *Arabidopsis* (Moran and Thompson 2001).

On the other hand, there is plenty of evidence that indicates the participation of jasmonic acid (JA) or its derivatives in triggering defence responses of different plants against herbivores, including spider mites (Li et al. 2002, 2004; Omer et al. 2001; Thaler et al. 2002; Bari and Jones 2009; Zhurov et al. 2014). One of the first steps in JA synthesis is oxygenation of alpha-linolenic acid with the participation of lipoxygenase (LOX). This enzyme is involved in many plant defence responses (Wasternack and Hause 2013). Elevated expression of the LOX gene has been observed in *T. urticae* infested plants (Miyazaki et al. 2014). It has also been found that exogenous jasmonic acid application causes an increase in the expression of the LOX gene (Porta et al. 1999; Yang et al. 2012).

Recent studies have shown that it is possible for both salicylic acid to participate in responses associated with pest infestation and for jasmonic acid to participate in defence responses associated with pathogen infection. It has been demonstrated that SA or BTH are involved in defence responses to phloem-feeding or piercing-sucking herbivores, e.g., against aphids on tomato (Boughton et al. 2006; Cooper et al. 2004) and two-spotted spider mites on beans and tomatoes (Choh et al. 2004; Favaro et al. 2019). On the other hand, jasmonic acid seems to be an effective elicitor of resistance to necrotrophic pathogens (Thaler et al. 2004). However, the use of resistance inducers in practice poses difficulties.

The complexity of the mechanism of resistance induction in plants is the reason why the effectiveness of this process cannot satisfy the expectations of growers. The results of the induction depend on many factors, including plant species, concentration of elicitor, or phenological stage of the plant. It has been found that nitrogen fertilization can reduce the level of secondary metabolites in plant tissues and cause increased susceptibility of plants to pathogens or pests (Koricheva 2002; Herms 2002; Throop and Lerdau 2004). The situation is complicated by the antagonistic interplay between JA and SA in plant defence response (Koornneef and Pieterse 2008; Smith et al. 2009; Gimenez-Ibanez and Solano 2013). This has been demonstrated in a series of herbivore-pathogen systems for model plants (Thaler et al. 2012).

The aim of the experiments reported in this paper was to investigate the impact of JA and BTH applied to apple trees grown under the conditions of optimal fertilization with nitrogen and under deficiency of this component on the population of the two-spotted spider mite. Additionally, we studied the mutual relationship between the applied defence elicitors by assessing the expression of the PAL and LOX genes encoding key enzymes involved in salicylic acid and jasmonic acid biosynthesis.

## Materials and methods

### Plant treatments

Experiments were performed in a greenhouse on 2-year-old apple trees cv. ‘Gala’ grafted on M.9, which were grown in 5 l containers filled with a mixture of sand and peat in a ratio of 4:1. The average number of leaves on the trees at the start of the experiments was 25. The experiments were used to study the impact of two factors: elicitor application and nitrogen fertilization. The elicitor factor was used in nine variants: 1.5 or 2.5 mM solution of JA (JA1, JA2); 0.5 or 1.5 mM solution of BTH (BTH1, BTH2); JA and BTH applied at concentrations of 1.5 and 0.5 mM (JA1BTH1) or 2.5 and 1.5 mM (JA2BTH2); controls for JA (1% acetone; KJA), BTH (water; KBTH) and untreated plants (K). Bion 50 WG was used as a source of BTH. The trees were sprayed with the experimental solutions using a hand sprayer. The fertilization factor appeared in two variants: deficient (N1) and optimum (N2) nitrogen fertilization.

Each tree was fertilized with 0.5 g of potassium sulfate (52% K_2_O) and 0.4 g of magnesium sulfate (16% MgO) every 2 weeks. In addition, at the same intervals, the trees of the N2 combination were fertilized with nitrogen at a dose of 0.15 g of ammonium sulfate (20.8% N). Phosphorus was added once in the amount of 1 g superphoshate (46% P_2_O_5_) per 5 L of the substrate. The above doses of applied nutrients and modes of their application corresponded with the fertilizer recommendations proposed by Wójcik (2018) for high-density apple orchards established on coarse-textured soil with a low status of both organic matter and available phosphorus, potassium and magnesium. Plants were irrigated depending on the moisture content of the substrate using a drip line system.

The trees treated in the above manner were used in the experiment on the influence of the elicitor and fertilization factors on the fecundity of the two-spotted spider mite and the development of its population. In a parallel experiment carried out on different apple trees, the expression of the PAL and LOX genes was analyzed.

### Development of *Tetranychus urticae* populations on potted apple trees

The day after treatment with elicitors, the plants were infested with *T. urticae* females that had been reared on bean plants. Ten specimens of two-spotted spider mite females were introduced onto each tree. After 4 weeks, all leaves from each tree were collected and the mites were brushed off using a mite-brushing machine (Henderson and McBurnie 1943). The mites (eggs as well as all other developmental stages together) were counted under a binocular microscope. Each of the experimental treatments was performed in 8 replicates (trees) and the entire experiment was performed in a completely randomized design.

### Fecundity test

The leaves for the fecundity test were taken from the same trees on which the development of the population of spider mites was analyzed. The fecundity test was performed in an environmental chamber at 22 ºC, 75% relative humidity, and a 16:8 h light/dark photoperiod. Plexiglas™ breeding cages (1 cm diameter) were placed on the leaves whose stalks were dipped in tubes filled with water. Two-day-old inseminated females of *T. urticae* reared on bean were placed separately in cages on the lower surface of the leaf. The eggs laid were counted after 3 days. Each of the experimental treatments was performed in 12 replicates (12 leaves from eight trees).

### RNA extraction and gene expression analysis

For gene expression analysis, three leaves were taken from the middle part of the shoot of each of the eight trees belonging to the experimental combination. The analyses were performed on pooled samples in three replications. Leaves were collected directly into liquid nitrogen, ground in liquid nitrogen, and stored at − 80 °C. The leaves were sampled 2 and 14 days after elicitor treatment. Total RNA was extracted from the leaf samples according to Chang et al. (1993) and then digested with RQ RNase-Free DNase (Promega) according to the manufacturer’s instructions. The RNA samples were purified from the reaction mixture using RNeasy Mini Kit (Qiagen) according to the protocol for RNA clean-up. Concentration and purity of the RNA was examined spectrophotometrically in triplicate and by electrophoresis in agarose gel. From each RNA sample, 1 µg was reverse-transcribed using M-MLV reverse transcriptase (Promega) and oligo(dT)_15_ primer (Promega) in a 25-µl reaction volume, and the obtained cDNA samples were used in gene expression analyses.

The analysis assessed the expression of the genes encoding the lipoxygenase (LOX) and the phenylalanine ammonia-lyase (PAL) enzymes. The gene expression analysis was performed using the quantitative real-time PCR technique. As a reference gene, the constitutively expressed 18 s rRNA gene was applied (Warabieda et al. 2015). Primers for *PAL*, *LOX* and 18 s rRNA qRT-PCR were designed based on apple cDNA sequences available in GenBank (NCBI, National Center for Biotechnology Information; http://www.ncbi.nlm.nih.gov). The accession numbers of sequences, sequences of primers and lengths of amplification products are presented in Table 1. Quantitative RT-PCR was carried out in Rotorgene 6000 (Corbett Research) using KAPA™ Sybr Fast qPCR Master Mix (Kapa Biosystems) in a total volume of 20 µl. Annealing temperature for all primers was 60 °C. The melting curves of the amplified products were analyzed at the end of each PCR, the analysis being carried out at 72–95 °C, with temperature raised by 1 °C/5 s. For the PCR efficiency calculation and generation of the standard curve (correlation coefficient > 0.99), four 10-fold dilutions of cDNA were run together with analyzed samples. All qRT-PCR reactions were done with three technical replicates. Amplification products were additionally analysed by electrophoresis in 2.5% agarose gel and commercially sequenced to confirm their identity/homology with respect to the original sequences. For relative quantification, the standard curve method was applied (Larionov et al. 2005). The relative mRNA level of *PAL* and *LOX* was normalized with respect to the 18 s rRNA reference gene. All calculations were done using the Rotor-Gene 6000 Series Software 1.7.

**Table 1:** Primers used in the study

Gene	Primer sequences (5′ → 3′)	Product size (bp)	GenBank number
*LOX*	Forward: TTCGGAACTCCAATCCTGGTGGAA Reverse: GTTGATTGCTGCATGATGGGCTGA	157	GO527734
*PAL*	Forward: TATCGACCCAATTCTGGGTTGCCT Reverse: ATGCAGCATGTAAACCGTGACGTG	85	X68126 MT819947
18S rRNA	Forward: CGCGCAAATTACCCAATCCTGACA Reverse: TTGCCCTCCAATGGATCCTCGTTA	125	DQ341382

The effect of elicitor treatment on the LOX and PAL gene expression levels was estimated by comparison with the expression levels of these genes in control plants. For the JA and simultaneous JA and BTH (JABTH) treatments, the comparison was done relative to the KJ control (1% acetone), for the BTH treatments—relative to the KBTH control (water). The incidence of mutual relations between JA and BTH (antagonism, synergism, or lack of relation) was estimated. The influence of JA on BTH was assessed by comparing gene expression in plants after the concomitant use of both elicitors (JABTH) with the expression of the genes after the application of BTH alone. The effect of BTH on JA was determined by comparing gene expression in the plants treated with JABTH *versus* in those treated with JA alone.

### Statistical analysis

The results of the experiments were subjected to a two-way ANOVA (elicitor × N-fertilization). For the stabilization of variance, logarithmic or Box-Cox transformation (Osborne 2010) was used when necessary. The means were separated using Duncan’s multiple range test at α = 0.05. Statistical analysis was performed with the STATISTICA v.13 program (StatSoft, Tulsa, OK, USA).

## Results

### Fecundity test

**Fig. 1:**
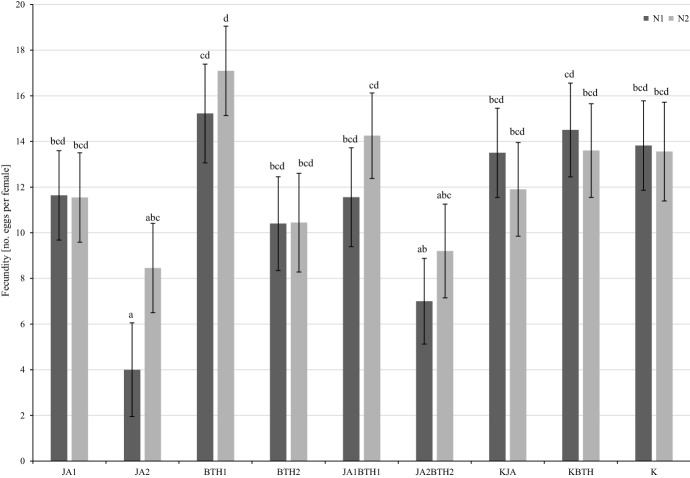
Influence of elicitors and N-fertilization on the mean (± SE; n = 12) fecundity of *Tetranychus urticae* females. Means capped with the same letter do not differ significantly (two-way ANOVA followed by Duncan’s test: p > 0.05). JA1, 1.5 mM solution of JA; JA2, 2.5 mM solution of JA; BTH1, 0.5 mM solution of BTH; BTH2, 1.5 mM solution of BTH; JA1BTH1, JA and BTH applied at 1.5 and 0.5 mM, respectively; JA2BTH2, JA and BTH applied at 2.5 and 1.5 mM, respectively; KJA, control for JA (1% acetone); KBTH, control for BTH (water); K, control (plants not treated); N1, N-deficient fertilization, N2, N-optimal fertilization

Two-way ANOVA revealed that the application of elicitors had a significant impact on the fertility of female spider mites (F_8,168_ = 4.67, p < 0.001). On the other hand, the N-fertilization factor was non-significant (F_1,168_ = 0.96, p = 0.33). The least number of eggs laid by females of *T. urticae* was found on the leaves treated with jasmonic acid at 2.5 mM. This was particularly pronounced in the case of the leaves collected from N-deficient trees, wherein the average number of eggs observed after 3 days of the experiment was 4 (Fig. 1). There were no significant differences between the fecundity of *T. urticae* females feeding on the leaves treated with all the other solutions of elicitors in comparison with the control combinations.

**Fig. 2:**
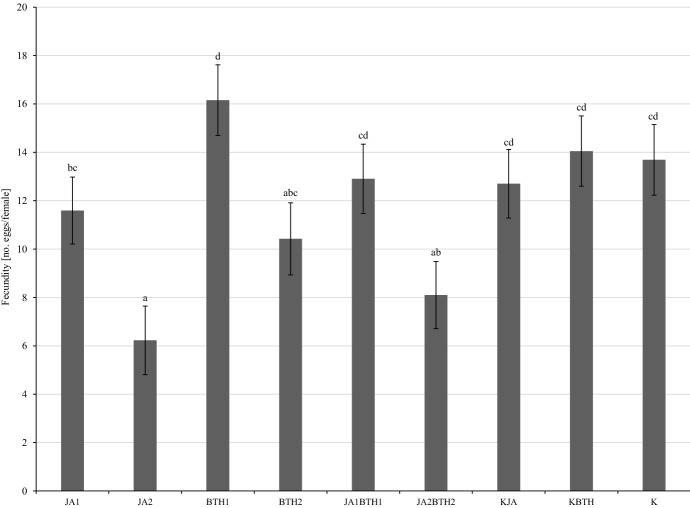
Influence of elicitors on the mean (± SE; n = 12) fecundity of *Tetranychus urticae* females. Means capped with the same letter do not differ significantly (two-way ANOVA followed by Duncan’s test: p > 0.05). JA1, 1.5 mM solution of JA; JA2, 2.5 mM solution of JA; BTH1, 0.5 mM solution of BTH; BTH2, 1.5 mM solution of BTH; JA1BTH1, JA and BTH applied at 1.5 and 0.5 mM, respectively; JA2BTH2, JA and BTH applied at 2.5 and 1.5 mM, respectively; KJA, control for JA (1% acetone); KBTH, control for BTH (water); K, control (plants not treated)

Because the two-way ANOVA revealed that the interaction of elicitor × N-fertilization was not significant (F_8,168_ = 0.48, p = 0.87), in order to better illustrate the relationship between the elicitors and fecundity of the mites, the means for this experimental factor were presented in a graph. It showed that the smallest number of eggs was found on the leaves treated with JA2 (Fig. 2). The addition of BTH at the higher concentration resulted in some increase in the fecundity of female mites on apple leaves (treatment with JA2BTH2 vs. JA2); however, the difference was not significant. The same situation was observed for the JA1 and JA1BTH1 treatments. On the other hand, the highest number of eggs was laid by mites on the leaves treated with the BTH1 solution (Fig. 2).

### Influence of elicitors on the development of *Tetranychus urticae* populations

**Fig. 3:**
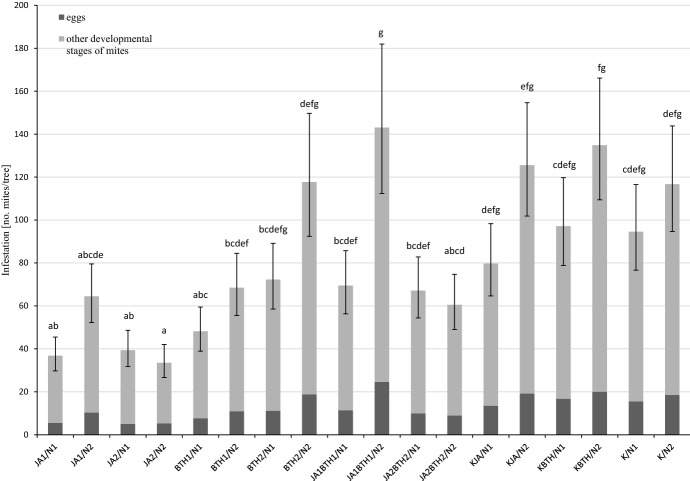
Influence of elicitors and N-fertilization on the mean (± SE; n = 08) population size of *Tetranychus urticae* infesting apple trees. Means capped with the same letter do not differ significantly (two-way ANOVA followed by Duncan’s test: p > 0.05). JA1, 1.5 mM solution of JA; JA2, 2.5 mM solution of JA; BTH1, 0.5 mM solution of BTH; BTH2, 1.5 mM solution of BTH; JA1BTH1, JA and BTH applied at 1.5 and 0.5 mM, respectively; JA2BTH2, JA and BTH applied at 2.5 and 1.5 mM, respectively; KJA, control for JA (1% acetone); KBTH, control for BTH (water); K, control (plants not treated); N1, N-deficient fertilization, N2, N-optimal fertilization

**Fig. 4:**
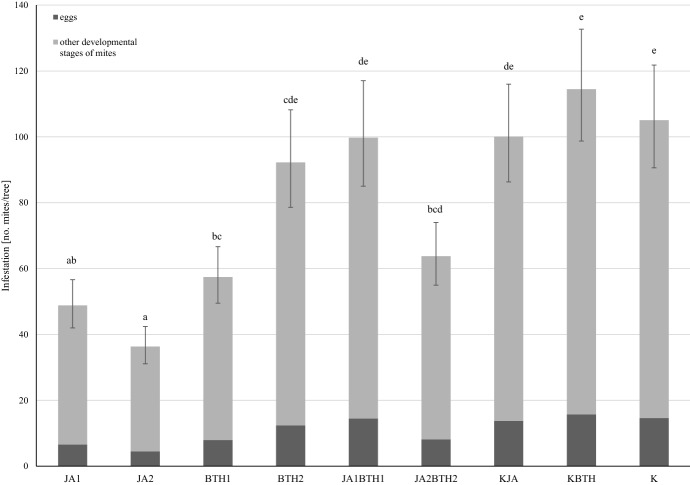
Influence of elicitors on the mean (± SE; n = 08) population size of *Tetranychus urticae* infesting apple trees. Means capped with the same letter do not differ significantly (two-way ANOVA followed by Duncan’s test: p > 0.05). JA1, 1.5 mM solution of JA; JA2, 2.5 mM solution of JA; BTH1, 0.5 mM solution of BTH; BTH2, 1.5 mM solution of BTH; JA1BTH1, JA and BTH applied at 1.5 and 0.5 mM, respectively; JA2BTH2, JA and BTH applied at 2.5 and 1.5 mM, respectively; KJA, control for JA (1% acetone); KBTH, control for BTH (water); K, control (plants not treated)

The treatment of plants with the elicitors had a significant impact on the development of spider mite populations in the experiment carried out on the potted plants (F_8,121_ = 7.09, p < 0.001). Four weeks after spraying the trees with the elicitors, the least number of mites was found on the trees treated with jasmonic acid. This was particularly evident in the case of JA1 applied to the N-deficient trees and JA2-treated trees grown at both levels of N-fertilization (Fig. 3). In most cases, a lower number of spider mites was found on the trees sprayed with JA only than on the trees treated simultaneously with JA and BTH at the corresponding concentrations. This was confirmed statistically for JA1 vs. JA1BTH1 for the trees fertilized with nitrogen. As the interaction elicitor x N-fertilization was not significant (F_8,121_ = 0.91, p = 0.51), the size of the spider mite population was also analysed taking into account only the main effect ‘elicitor’. Significantly more spider mites were found on the trees treated with JA + BTH compared to the trees treated only with JA (Fig. 4). This indicates the inhibitory effect of jasmonic acid on the pest population and the weakening of this effect by BTH.

N-fertilization had a significant effect on the development of two-spotted spider mite populations (F_1,121_ = 9.83, p = 0.002). The mean number of spider mites was higher on N-fertilized plants (86.7) than on N-deficient plants (63.7). The highest efficacy, according to Abbott’s (1925) formula, in limiting *T. urticae* populations was obtained in the case of plant treatment with JA2, with the highest level achieved by applying JA2 to N-fertilized trees (Table 2).

**Table 2:** Efficacy of elicitors in limiting the number of two-spotted spider mites on N-deficient and N-optimal apple trees according to Abbott’s formula: % efficacy = (1 – T/C) * 100, where T = number of mites on the treated plants and C = number of mites on the untreated plants

Apple trees	JA1	JA2	BTH1	BTH2	JA1 + BTH1	JA2 + BTH2
N-deficient (N1)	61.0^a^	58.4	49.1	23.5	26.5	29.0
N-optimal (N2)	44.7	71.3	41.3	− 0.8	− 22.5	48.1

^a^In relation to untreated trees (K)

### Gene expression

#### LOX gene expression

At the first sampling time-point, 2 days after treatment, elicitor (F_8,36_ = 9.63, p < 0.001) and N-fertilization (F_1,36_ = 227.45, p < 0.001) had significant effects, as well as their interaction (F_8,36_ = 13.54, p < 0.01). Similarly, 14 days after treatment, elicitor (F_8,36_ = 2.61, p < 0.05) and N-fertilization (F_1,36_ = 2.39, p < 0.001) had significant effects, as well as their interaction (F_8,36_ =4.12, p < 0.01).

**Fig. 5:**
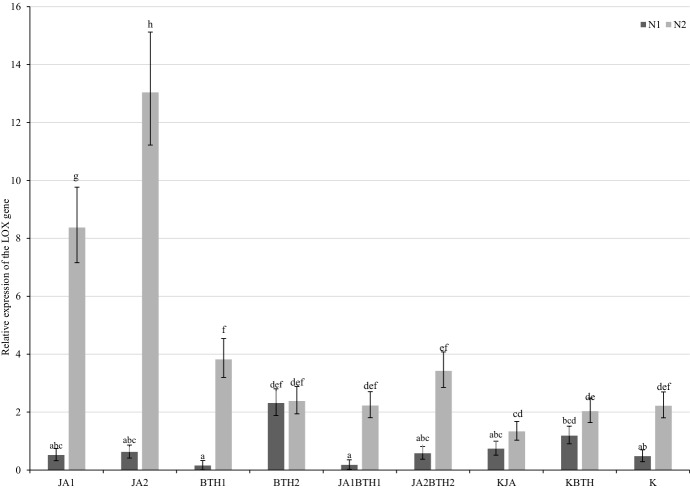
Mean (± SE; n = 03) relative expression of the LOX gene 2 days after elicitor treatment. Means capped with the same letter do not differ significantly (two-way ANOVA followed by Duncan’s test: p > 0.05). JA1, 1.5 mM solution of JA; JA2, 2.5 mM solution of JA; BTH1, 0.5 mM solution of BTH; BTH2, 1.5 mM solution of BTH; JA1BTH1, JA and BTH applied at 1.5 and 0.5 mM, respectively; JA2BTH2, JA and BTH applied at 2.5 and 1.5 mM, respectively; KJA, control for JA (1% acetone); KBTH, control for BTH (water); K, control (plants not treated); N1, N-deficient fertilization, N2, N-optimal fertilization

**Fig. 6:**
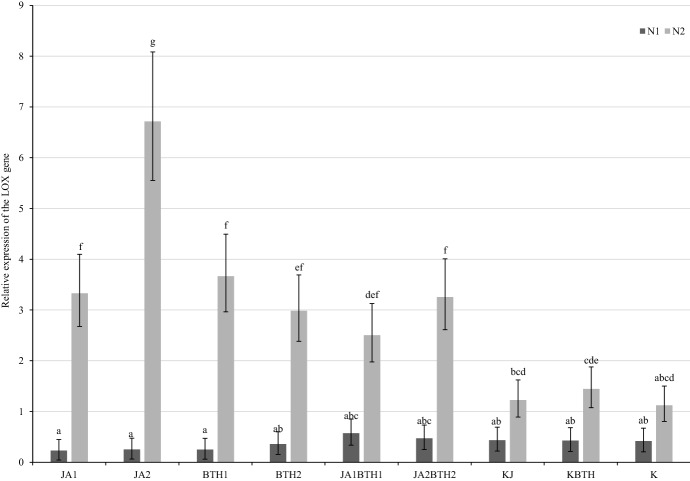
Mean (± SE; n = 03) relative expression of the LOX gene 14 days after elicitor treatment. Means capped with the same letter do not differ significantly (two-way ANOVA followed by Duncan’s test: p > 0.05). JA1, 1.5 mM solution of JA; JA2, 2.5 mM solution of JA; BTH1, 0.5 mM solution of BTH; BTH2, 1.5 mM solution of BTH; JA1BTH1, JA and BTH applied at 1.5 and 0.5 mM, respectively; JA2BTH2, JA and BTH applied at 2.5 and 1.5 mM, respectively; KJA, control for JA (1% acetone); KBTH, control for BTH (water); K, control (plants not treated); N1, N-deficient fertilization, N2, N-optimal fertilization

Generally, the expression of *LOX* was much higher in the plants growing in N-optimal conditions. In N-deficient ‘Gala’ trees, no increase in *LOX* expression as a result of JA and/or BTH treatments was detected at both sampling time-point (Figs. 5 and 6). Up-regulation of *LOX* was observed in optimally N-fertilized ‘Gala’ trees 2 days after spraying with 1.5 and 2.5 mM JA (6.3- and 9.8-fold increse in relative expression in comparison with KJA plants, respectively). The effect decreased with time; 14 days after JA application, *LOX* expression was 2.7- and 5.5-fold higher than in the control in the plants sprayed with 1.5 and 2.5 mM JA, respectively.

The treatments with BTH also affected the level of *LOX* mRNA, but the effect was weak and was significant only in the plants sprayed with 0.5 mM BTH, both 2 and 14 days after treatment. Simultaneous application of JA and BTH on ‘Gala’ apple trees did not markedly change the *LOX* expression pattern. A significant increase in the *LOX* mRNA levels relative to the control was observed only in the optimally N-fertilized plants treated with JA2BTH2 (2.6- and 2.7-fold increase, 2 and 14 days after spaying, respectively; Figs. 5 and 6).

### Mutual relationship between JA and BTH in the induction of LOX gene expression

#### Influence of JA on BTH (BTH vs. JABTH treatments)

In the case of trees growing under nitrogen deficiency, 2 days after elicitor application, the expression of *LOX* in the plants treated with JA1BTH1 was found to be similar to the expression of this gene after treatment with BTH1 alone. In contrast, LOX gene expression was almost 4-fold lower after treatment with JA2BTH2 than after spraying with BTH2 alone (Fig. 5). The expression of the LOX gene observed 14 days after the application of BTH was not affected by JA regardless of the concentration of the elicitors (Fig. 6).

For the trees growing in the substrate with the optimum level of nitrogen, there were no significant differences in the expression of *LOX* between the plants treated with BTH1 and JA1BTH1, nor between those treated with BTH2 and JA2BTH2.

#### Influence of BTH on JA (JA vs. JABTH treatments)

In the trees optimally fertilized with nitrogen, 2 days after treatments, the addition of BTH (JA1BTH1 and JA2BTH2) significantly reduced the expression of the LOX gene compared to the expression of this gene in the plants treated with the corresponding JA solutions alone. The same effect was observed 14 days after treatment, but only in the case of JA2 vs. JA2BTH2. For N-deficient trees, no significant effect of BTH on JA with regard to LOX gene expression was observed at either sampling time-point (Figs. 5 and 6).

Generally, as far as the mutual relations between JA and BTH in the induction of LOX gene expression are concerned, there was a more frequent occurrence of an antagonistic influence of BTH on JA (three cases) than of JA on BTH (one case). This negative effect of BTH on JA was particularly evident 2 days after treatment on the trees fertilized with nitrogen. It should be noted that no synergy between JA and BTH in influencing LOX gene expression was found.

### PAL gene expression

The effects of the two experimental factors on PAL gene expression were significant for both observation dates. At the first sampling time-point, elicitor (F_8,36_ = 11.10, p < 0.001) and N-fertilization (F_1,36_ = 159.84, p < 0.001) had significant effects, as well as their interaction (F_8,36_ = 10.49, p < 0.01). Similarly, 14 days after treatment, the effect of elicitor (F_8,36_ = 37.08, p < 0.01) and N-fertilization (F_1,36_ = 68.40, p < 0.001) were significant, just as that of their interaction (F_8,36_ = 34.69, p < 0.01).

**Fig. 7:**
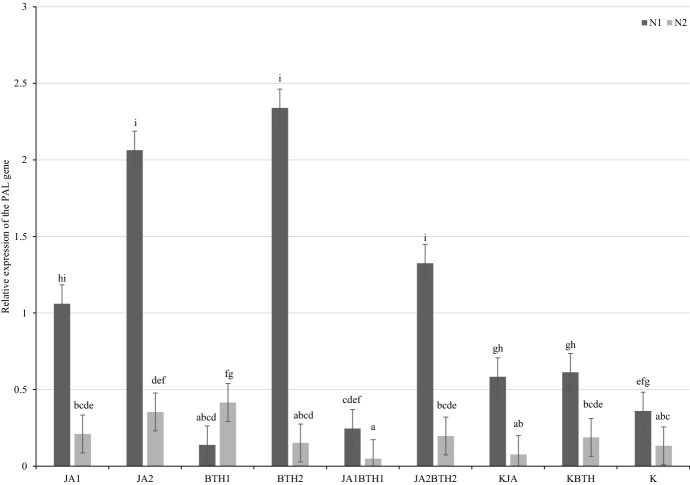
Mean (± SE; n = 03) relative expression of the PAL gene 2 days after elicitor treatment. Means capped with the same letter do not differ significantly (two-way ANOVA followed by Duncan’s test: p > 0.05). JA1, 1.5 mM solution of JA; JA2, 2.5 mM solution of JA; BTH1, 0.5 mM solution of BTH; BTH2, 1.5 mM solution of BTH; JA1BTH1, JA and BTH applied at 1.5 and 0.5 mM, respectively; JA2BTH2, JA and BTH applied at 2.5 and 1.5 mM, respectively; KJA, control for JA (1% acetone); KBTH, control for BTH (water); K, control (plants not treated); N1, N-deficient fertilization, N2, N-optimal fertilization

Two days after treatment, in N-deficient trees sprayed with JA2, BTH2 or JA2BTH2, a significant increase in the *PAL* mRNA level was found in comparison with the control treatments KJA, KBTH, and KJA. In the trees sprayed with BTH1 and JA1BTH1, a slight reduction in the relative expression of this gene in relation to the control trees was observed. On the other hand, in the N-optimal trees, a significant increase in *PAL* expression was found in the trees treated with JA2 and BTH1 (Fig. 7). Generally, at this sampling time-point, the expression of the PAL gene was higher in N-deficient trees than in N-optimal trees.

**Fig. 8:**
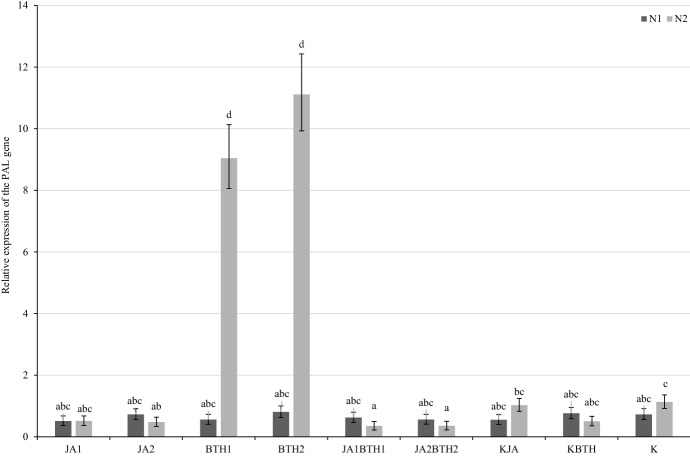
Mean (± SE; n = 03) relative expression of the PAL gene 14 days after elicitor treatment. Means capped with the same letter do not differ significantly (two-way ANOVA followed by Duncan’s test: p > 0.05). JA1, 1.5 mM solution of JA; JA2, 2.5 mM solution of JA; BTH1, 0.5 mM solution of BTH; BTH2, 1.5 mM solution of BTH; JA1BTH1, JA and BTH applied at 1.5 and 0.5 mM, respectively; JA2BTH2, JA and BTH applied at 2.5 and 1.5 mM, respectively; KJA, control for JA (1% acetone); KBTH, control for BTH (water); K, control (plants not treated); N1, N-deficient fertilization, N2, N-optimal fertilization

Fourteen days after treatment, there was a clear increase in PAL gene expression in the trees sprayed with BTH at both concentrations, and, in contrast to the first sampling time-point, it concerned only the N-optimal plants (Fig. 8); the level of *PAL* mRNA was 18- and 22.1-fold higher in the plants sprayed with BTH1 and BTH2, respectively, as compared to KBTH control plants. In the other experimental combinations, no increase in *PAL* expression was detected at this sampling time-point.

### Mutual relationship between JA and BTH in the induction of PAL gene expression

#### Influence of JA on BTH (BTH vs. JABTH treatments)

As far as PAL gene expression is concerned, the most evident negative effect of JA on BTH treatments was found 14 days after elicitors application in the leaves of N-optimal trees and at both levels of elicitor concentrations (BTH1 vs. JA1BTH1, and BTH2 vs. JA2BTH2) (Fig. 8). Two days after elicitors application, the same effect was detected in the case of N-optimal trees treated with the lower concentrations of the elicitors (BTH1 vs. JA1BTH1) (Fig. 7).

#### Influence of BTH on JA (JA vs. JABTH treatments)

The antagonistic effect of BTH vs. JA on the PAL expression level was only found 2 days after elicitors application in the apple leaves sprayed with the lower concentrations of the elicitors (JA1 vs. JA1BTH1), regardless of the nitrogen supply (Fig. 7).

In general, as far as the interaction of JA and BTH on PAL gene expression is concerned, the negative effect of BTH on JA was found in two cases, whereas that of JA on BTH in three cases. No synergistic effect of the BTH and JA treatments on *PAL* up-regulation was found.

Generally, treatment of young ‘Gala’ apple trees with JA resulted in up-regulation of the LOX gene, and treatment with BTH resulted in up-regulation of the PAL gene, as compared to the control. The induction of gene expression in the elicitor-treated trees was more pronounced in the plants regularly fertilized with nitrogen. The inducing effect on *LOX* and *PAL* expression observed in the plants sprayed with JA and BTH applied separately was eliminated in the plants treated simultaneously with JA and BTH, which indicates antagonism between the two elicitors.

## Discussion

The mutual relationships between jasmonic acid, BTH and N-fertilization in the induction of resistance mechanisms in apple trees was analysed by determining the spider mite performance as well as the expression of selected genes. The leaves collected for real-time PCR analyses came from trees not infested by the pest. We wanted to check how the tested elicitors change the expression of genes that may be related to resistance against the two-spotted spider mite. If we had analysed the leaves inhabited by the spider mite, we would not be sure whether the changes in gene expression were the result of elicitors treatment or spider mites feeding. It is known that pests can induce plant resistance mechanisms in a similar, but not exactly the same way as elicitors can (Dicke et al. 1999). An open question is how the studied elicitors and spider mite feeding interact in the process of inducing the expression of various apple genes.

On the other hand, in experiments where the influence of applied elicitors on the fecundity and growth of spider mite population was studied, changes in mite performance were proven. This must have been related to the induction of immunity in apple trees as there is no evidence that jasmonic acid or acibenzolar-*S*-methyl has a direct toxic effect on spider mites.

The final population size of the *T. urticae* in the greenhouse experiment depended on the plant’s defence response that occurred during the entire 4-week period of the experiment. As the response of the plant changes over time, the analysis of LOX and PAL gene expression was therefore performed in our study at two time points, namely 2 and 14 days after elicitor application. On the other hand, the results of the 3-day fecundity test could be related only to the results of genes expression determined 2 days after elicitor application.

In contrast to the fecundity test, in the greenhouse experiment, not only the number of eggs, but also all developmental stages of the two-spotted spider mite were considered together. This approach allows a more reliable assessment of the influence of experimental factors on the reproductive potential of the mite, as the proportions of its various developmental stages change over time.

The results of the fecundity test as well as those of the experiment conducted on potted apple trees indicated the important role of jasmonic acid in the induction of defence responses against *T. uticae*. The treatment of trees with a solution of jasmonic acid, especially at the higher concentration, diminished the performance of two-spotted spider mites (Figs. 1, 2, 3 and 4). The results obtained in both experiments are in agreement with the generally accepted opinion concerning the important role of jasmonic acid in triggering defence responses against herbivores (Rohwer and Erwin 2010). The negative effect of exogenous jasmonic acid on spider mite performance has been found in a number of studies on plants of different species (Thaler et al. 2002; Omer et al. 2001; Choh et al. 2004; Kawazu et al. 2012; Warabieda and Olszak 2010).

In our study, the importance of jasmonic acid in the induction of defence responses was also indirectly confirmed by up-regulation of the LOX gene that was observed in the leaves of the trees treated with JA and fertilized with nitrogen. This was particularly unambiguous 2 days after spraying (Figs. 5 and 6). The LOX gene belongs to the early response genes of octadecanoid pathway enzymes, as proved by other authors (Li et al. 2004; Heitz et al. 1997). The LOX enzyme is crucial in the regulation of biosynthesis of jasmonates and, therefore, it is important in many immune responses, including plant resistance to spider mites (Wasternack and Hause 2013; Miyazaki et al. 2014). Our results seem to confirm the thesis that jasmonic acid induces the expression of *LOX*, which may be the cause of positive feedback and induction of the biosynthesis of JA, i.e., of itself (Feng et al. 2007; Xu et al. 2005).

Analyzing the mutual relationships between the elicitors in the induction of LOX gene expression, there were more cases of an antagonistic effect of BTH on JA than vice versa, i.e., JA on BTH. This antagonistic influence of BTH on JA was also evident in the potted plants in the greenhouse experiment. In general, the number of mites on the trees treated with JA + BTH was higher than on the trees treated with JA only. On the trees fertilized with nitrogen and treated with solutions of the elicitors at their lower concentrations, significantly more spider mites were observed on the JA1BTH1-sprayed trees in comparison with the trees treated exclusively using the lower concentration of JA (JA1) (Fig. 3). When the higher concentrations of the elicitors were used (JA2BTH2 and JA2), the situation was similar, but the difference was not significant. Perhaps the jasmonic acid concentration factor turned out too influential in this case, masking the antagonistic effect of BTH. The possible effects of the concentrations of elicitors used simultaneously on resistance induction have been reported previously (Thaler et al. 2002; Mur et al. 2006). It is worth noting that, in the fecundity test, the inhibitory effect of BTH on resistance induction in apple by JA was not statistically proven. Perhaps it was influenced by the short duration of this experiment.

Some studies indicate the possibility of inducing plant resistance to pests using SA or BTH. This was demonstrated in pests with a piercing and sucking mouth apparatus, for example peach aphid (*Myzus persicae*) and potato aphid (*Macrosiphum euphorbiae*) on tomato (Boughton et al. 2006; Cooper et al. 2004). In the present study, the effect of BTH application on the fecundity of *T. urticae* females in the laboratory experiment was not very well pronounced. On the other hand, in the experiment with potted plants, fewer mites were found on the trees treated with the BTH solution at the lower concentration than in the control plants (KBTH) (Figs. 3 and 4). Furthermore, a significant difference was found when the statistical analysis was carried out only for the elicitor factor (Fig. 4). It should be pointed out that in the case of the trees treated with BTH, the *T. urticae* mites were more abundant than on the trees treated with jasmonic acid, which was clearly evident after using the higher concentration of this compound. This indicates that JA, rather than BTH, has a significant role in the induction of apple resistance to mites. This had also been the conclusion of other authors, e.g., in an experiment carried out on bean plants infested with two-spotted spider mites, in which the tested compounds were applied to the soil (Choh et al. 2004). Similarly, a study conducted on apple trees in open field conditions demonstrated that although BTH restricted the population of the European red mite (*Panonychus ulmi* Koch), it was less effective than methyl jasmonate, a derivative of jasmonic acid (Warabieda 2015).

With respect to the PAL gene, the expression of which was studied in the present experiment, it should be emphasized that PAL, which is a key enzyme in the synthesis of salicylic acid and plays an important role in SA-dependent defence to control pathogens, may also be important in the defence responses against herbivores. In an experiment conducted on Arabidopsis, treatment of plants with BTH had not influenced PAL expression within the first 3 days; however, expression of this gene significantly increased after subsequent infection caused by *Pseudomonas syringae* pv. tomato (Kohler et al. 2002). In contrast, an unambiguous determination of the impact of the elicitors used in our study on PAL gene expression was hampered because of its high variability depending on the sampling time-point and N-fertilization level (Figs. 7 and 8). It is noteworthy that the high increase in relative PAL gene expression in nitrogen-fertilized trees that was observed 14 days after spraying them with BTH solutions (Fig. 8) was not reflected in the parallel potted experiment. Although fewer spider mites were found on the trees treated with BTH than on the control trees (KBTH), the differences were not significant (Fig. 3).

Regarding the mutual relationship between BTH and JA in the induction of PAL gene expression, it was found that 14 days after treatments significantly lower expression of the PAL gene was observed on the plants treated concomitantly with JA + BTH used at the two tested concentrations, compared to the plants treated only with BTH. This may confirm the existence of antagonism between JA and BTH in the induction of the signal pathway associated with salicylic acid, for which PAL is an essential element (Fig. 8).

It seems that due to the large variability in PAL gene expression, its measurement is not a good marker of apple resistance to spider mites or other pests. Similar conclusions had been put forward by Putthof et al. (2010) in their studies on tomato, in which they observed an increase in the expression of PAL after the whiteflies *Bemisia tabaci* and *Trialeurodes vaporariorum* were allowed to feed on the plants, but the level of expression of this gene was different depending on the experiment.

Our research indicates a stimulating role of nitrogen fertilization on the development of spider mite populations, which was particularly evident in the greenhouse experiment. In the fecundity test, the results were less meaningful, which could have been caused by too short a duration of the experiment. However, our results are a confirmation of other studies that indicate a stimulatory effect of nitrogen on the growth of spider mite populations (Suski and Badowska 1975; Wermelinger et al. 1985; Chow et al. 2009). This may result from a reduction in the level of chemical compounds associated with the constitutive defence of the plant (Herms 2002; Hol et al. 2003; Orians et al. 2003; Chen et al. 2008). On the other hand, there have been studies indicating that nitrogen fertilization can cause an increase in induced defenses of plants against pests (Glynn et al. 2003; Cipollini and Bergelson 2001). This phenomenon is confirmed in our research. Although nitrogen fertilization stimulated the development of spider mite populations, the induction of resistance mechanisms was more effective in these plants. It was particularly pronounced in the plants after treatment with JA, which resulted in LOX gene up-regulation. A similar situation, but only at the second observation time-point, was found for the PAL gene after application of BTH, but this was not clearly related to pest abundance. However, the increase in PAL gene expression may be related to enhanced resistance to pathogens, which had been observed in ‘Gala’ trees in our previous study, in which significant induction of PR apple genes (PR-1a, PR-2, PR-3) was found after treatment with BTH (Warabieda et al. 2015). Similarly, after application of SA, increased activity of defence-related enzymes, including PAL, as well as elevated expression of five pathogenesis-related genes PR1, PR5, PR8, chitinase and β-1,3-glucanase were observed (Zhang et al. 2016).

Considering the potential use of the tested elicitors in limiting the numbers of the two-spotted spider mite, attention should be paid to their effectiveness. Our study indicates that out of the experimental treatments applied, the highest efficacy was obtained after the use of the higher concentration of jasmonic acid solution (JA2). However, in no case did the effectiveness of this elicitor exceed 80%. So, we can talk here about pest reduction rather than pest control.

On the other hand, the BTH solutions reduced plant infestation to a lesser extent, and in some cases an increase in spider mite abundance was observed, which was reflected in a negative value of effectiveness. The results also indicated an antagonistic relationship between BTH and JA. The efficacy of JA + BTH solutions in pest control was lower than that of the solutions containing only JA. This may indicate the need for some precaution when using BTH against pathogens on plants colonized by spider mites. The possibility of using jasmonic acid in the protection of apple trees against spider mites is also supported by our earlier studies, in which treatments with JA did not affect the growth and yielding of apple trees (Warabieda et al. 2015).

Unlike BTH, which has found commercial application, the use of jasmonic acid or its derivatives is problematic since they are oily substances immiscible in water. This disadvantage was overcome by the development of a patented method for obtaining water-soluble salts. This has opened up the possibility of using jasmonic acid as plant activator also in combination with herbicides, pesticides, bioactive or biological seed treatment components, and semiochemicals (Ghasemi Pirbalouti et al. 2014; Marks 2012).

## Conclusions

Among the tested elicitors, jasmonic acid showed significantly higher effectiveness in inducing apple tree resistance to *T. urticae*, as compared to BTH. This was reflected both in the number of pests in the potted experiment and in the expression of the LOX gene. As far as the mutual relationship between the tested elicitors are concerned, an antagonism between JA and BTH was demonstrated. The antagonistic effect of BTH on JA may lead to decreased effectiveness of the jasmonic acid used to stimulate the resistance of apple trees to the two-spotted spider mite while using BTH against pathogens. The study showed that it was possible to reduce the population of the two-spotted spider mite on apple trees by using a solution of jasmonic acid as an elicitor of resistance.

## References

[CR1] Abbott WS (1925). A method of computing the effectiveness of an insecticide. J Econ Entomol.

[CR2] Ament K, Kant MR, Sabelis MW, Haring MA, Schuurink RC (2004). Jasmonic acid is a key regulator of spider mite-induced volatile terpenoid and methyl salicylate emission in tomato. Plant Physiol.

[CR3] Bari R, Jones JDG (2009). Role of plant hormones in plant defence responses. Plant Mol Biol.

[CR4] Bazzi C, Biondi E, Berardi R, Brunelli A (2006). Efficacy of bioagents and chemicals against pear shoot blight. Acta Hortic.

[CR5] Boughton AJ, Hoover K, Felton GW (2006). Impact of chemical elicitor applications on greenhouse tomato plants and population growth of the green peach aphid, *Myzus persicae*. Entomol Exp Appl.

[CR6] Campos-Vargas R, Saltveit ME (2002). Involvement of putative chemical wound signals in the induction of phenolic metabolism in wounded lettuce. Physiol Plant.

[CR7] Chang S, Puryear J, Cairney J (1993). A simple and efficient method for isolating RNA from pine trees. Plant Mol Biol Rep.

[CR8] Chen Y, Schmelz EA, Wäckers F, Ruberson JR (2008). Cotton plant, *Gossypium hirsutum* L., defense in response to nitrogen fertilization. J Chem Ecol.

[CR9] Choh Y, Ozawa R, Takabayashi J (2004). Effects of exogenous Jasmonic acid and benzo (1,2,3) thiadiazole-7carbothioic acid S-methyl ester (BTH), a functional analogue of salicylic acid, on the egg production of herbivorous mite *Tetranychus urticae* (Acari: Tetranychidae). Appl Entomol Zool.

[CR10] Chow A, Chau A, Heinz KM (2009). Reducing fertilization for cut roses: effect on crop productivity and two spotted spider mite abundance, distribution, and management. J Econ Entomol.

[CR11] Cipollini DF, Bergelson J (2001). Plant density and nutrient availability constrain constitutive and wound-induced expression of trypsin inhibitors in *Brassica napus*. J Chem Ecol.

[CR12] Cooper WC, Jia L, Goggin FL (2004). Acquired and R-gene mediated resistance against the potato aphid in tomato. J Chem Ecol.

[CR13] Dehghan S, Sadeghi M, Pöppel A, Fischer R, Lakes-Harlan R, Kavousi HR, Vilcinskas A, Rahnamaeian M (2014). Differential inductions of phenylalanine ammonia-lyase and chalcone synthase during wounding, salicylic acid treatment, and salinity stress in safflower, *Carthamus tinctorius*. Biosci Rep.

[CR14] Dicke M, Gols R, Ludeking D, Posthumus M (1999). Jasmonic acid and herbivory differentially induce carnivore-attracting plant volatiles in lima bean plants. J Chem Ecol.

[CR15] Favaro R, Resende J, Gabriel A, Zeist AR, Cordeiro ECN, Favaro Júnior JL (2019). Salicylic acid: resistance inducer to two-spotted spider mite in strawberry crop. Hortic Bras.

[CR16] Feng YJ, Wang JW, Luo SM (2007). Effects of exogenous jasmonic acid on concentrations of direct-defense chemicals and expression of related genes in Bt (*Bacillus thuringiensis*) corn (*Zea mays*). Agric Sci China.

[CR17] Ghasemi Pirbalouti A, Sajjadi SE, Parang K (2014). A review (research and patents) on jasmonic acid and its derivatives. Arch Pharm.

[CR18] Gimenez-Ibanez S, Solano R (2013). Nuclear jasmonate and salicylate signaling and crosstalk in defense against pathogens. Front Plant Sci.

[CR19] Glynn C, Herms DA, Egawa M, Hansen R, Mattson WJ (2003). Effects of nutrient availability on biomass allocation as well as constitutive and rapid induced herbivore resistance in poplar. Oikos.

[CR20] González-Candelas L, Alamar S, Sánchez-Torres P, Zacarías L, Marcos JF (2010). A transcriptomic approach highlights induction of secondary metabolism in citrus fruit in response to *Penicillium digitatum* infection. BMC Plant Biol.

[CR21] Heitz T, Bergey DR, Ryan CA (1997). A gene encoding a chloroplast-targeted lipoxygenase in tomato leaves is transiently induced by wounding, systemin, and methyl jasmonate. Plant Physiol.

[CR22] Henderson CF, McBurnie HV (1943). Sampling techniques for determining populations of the citrus red mites and its predators. US Dept Agric Circ.

[CR23] Herms DA (2002). Effects of fertilization on insects resistance of woody ornamental plants: reassessing an entrenched paradigm. Environ Entomol.

[CR24] Hol WHG, Vrieling K, van Veen JA (2003). Nutrients decrease pyrrolizidine alkaloid concentrations in *Senecio jacobaea*. New Phytol.

[CR25] Kawazu K, Mochizuki A, Sato Y, Sugeno W, Murata M, Seo S, Mitsuhara I (2012). Different expression profiles of jasmonic acid and salicylic acid inducible genes in the tomato plant against herbivores with various feeding modes. Arthropod Plant Interact.

[CR26] Kohler A, Schwindling S, Conrath U (2002). Benzothiadiazole-induced priming for potentiated responses to pathogen infection, wounding, and infiltration of water into leaves requires the *NPR1/NIM1* gene in Arabidopsis. Plant Physiol.

[CR27] Koornneef A, Pieterse CMJ (2008). Cross talk in defense signaling. Plant Physiol.

[CR28] Koricheva J (2002). Meta-analysis of sources of variation in fitness costs of plant antiherbivore defenses. Ecology.

[CR29] Larionov A, Krause A, Miller W (2005). A standard curve based method for relative real time PCR data processing. BMC Bioinformatics.

[CR30] Li C, Williams MM, Loh YT, Lee GI, Howe GA (2002). Resistance of cultivated tomato to cell content-feeding herbivores is regulated by the octadecanoid-signaling pathway. Plant Physiol.

[CR31] Li L, Zhao Y, McCaig BC, Wingerd BA, Wang J, Whalon ME, Pichersky E, Howe GA (2004). The tomato homolog of coronatine-insensitive1 is required for the maternal control of seed maturation, jasmonate-signaled defense responses, and glandular trichome development. Plant Cell.

[CR32] Marks D (2012) Agricultural compositions containing a jasmonic acid or dihydrojasmonic acid salt. European Patent EP1740045

[CR33] Maxson-Stein K, He SY, Hammerschmidt R, Jones AL (2002). Effect of treating apple trees with acibenzolar-S-methyl on fire blight and expression of pathogenesis-related protein genes. Plant Dis.

[CR34] Miyazaki J, Stiller WN, Truong TT, Xu Q, Hocart CH, Wilson LJ, Wilson IW (2014). Jasmonic acid is associated with resistance to twospotted spider mites in diploid cotton (*Gossypium arboreum*). Funct Plant Biol.

[CR35] Moran PJ, Thompson GA (2001). Molecular responses to aphid feeding in Arabidopsis in relation to plant defense pathways. Plant Physiol.

[CR36] Mur LAJ, Kenton P, Atzorn R, Miersch O, Wasternack C (2006). The outcomes of concentration-specific interactions between salicylate and jasmonate signaling include synergy, antagonism, and oxidative stress leading to cell death. Plant Physiol.

[CR37] Norelli JL, Jones AL, Aldwickle HS (2003). Fire blight management in the twenty-first century: using new technologies that enhance host resistance in apple. Plant Dis.

[CR38] Nyaka Ngobisa AIC, Ntsomboh-Ntsefong G, Yun WM, Dzarifah MZ, Owona Ndongo PA (2016). Transcriptional expression of three putative pathogenesis-related proteins in leaves of rubber tree (*Hevea brasiliensis*) inoculated with *Neofusicoccum* ribis. J Appl Biol Biotechnol.

[CR39] Omer AD, Granett J, Karban R, Villa EM (2001). Chemically-induced resistance against multiple pests in cotton. Int J Pest Manage.

[CR40] Orians CM, Lower S, Fritz RS, Roche BM (2003). The effects of plant genetic variation and soil nutrients on secondary chemistry and growth in a shrubby willow, *Salix sericea*: patterns and constraints on the evolution of resistance traits. Biochem Syst Ecol.

[CR41] Osborne J (2010). Improving your data transformations: Applying Box-Cox transformations as a best practice. Pract Assess Res Eval.

[CR42] Pääkkönen E, Seppänen S, Holopainen T, Kokko H, Kärenlampi S, Kärenlampi L, Kangasjärvi J (1998). Induction of genes for the stress proteins PR-10 and PAL in relation to growth, visible injuries and stomatal conductance in birch (*Betula pendula*) clones exposed to ozone and/or drought. New Phytol.

[CR43] Porta H, Rueda-Benítez P, Campos F, Colmenero-Flores JM, Colorado JM, Carmona MJ, Covarrubias AA, Rocha-Sosa M (1999). Analysis of lipoxygenase mRNA accumulation in the common bean (*Phaseolus vulgaris* L.) during development and under stress conditions. Plant Cell Physiol.

[CR44] Puthoff DP, Holzer FM, Perring TM, Walling LL (2010). Tomato pathogenesis-related protein genes are expressed in response to *Trialeurodes vaporariorum* and *Bemisia tabaci* biotype B feeding. J Chem Ecol.

[CR45] Rohwer CL, Erwin JE (2010). Spider mites (*Tetranychus urticae*) perform poorly on and disperse from plants exposed to methyl jasmonate. Entomol Exp Appl.

[CR46] Sayari M, Babaeizad V, Ghanbari MAT, Rahimian H (2014). Expression of the pathogenesis related proteins, NH-1, PAL, and lipoxygenase in the iranian Tarom and Khazar rice cultivars, in reaction to *Rhizoctonia solani*: the causal agent of rice sheath blight. Journal of Plant Protection Research.

[CR47] Smith JL, De Moraes CM, Mescher MC (2009). Jasmonate- and salicylate-mediated plant defense responses to insect herbivores, pathogens and parasitic plants. Pest Manag Sci.

[CR48] Solecka D, Kacperska A (2003). Phenylpropanoid deficiency affects the course of plant acclimation to cold. Physiol Plant.

[CR49] Sparla F, Rotino L, Valgimigli MC, Pupillo P, Trost P (2004). Systemic resistance induced by benzothiadiazole in pear inoculated with the agent of fire blight (*Erwinia amylovora*). Sci Hortic.

[CR50] Suski ZW, Badowska T (1975). Effect of the host plant nutrition on the population of the two spotted spider mite, *Tetranychus urticae* Koch (Acarina, Tetranychidae). Ekologia Polska.

[CR51] Thaler JS, Fidantsef AL, Bostock RM (2002). Antagonism between jasmonate- and salicylate-mediated induced plant resistance: effects of concentration and timing of elicitation on defense-related proteins, herbivore, and pathogen performance in tomato. J Chem Ecol.

[CR52] Thaler JS, Owen B, Higgins VJ (2004). The role of the jasmonate response in plant susceptibility to diverse pathogens with a range of lifestyles. Plant Physiol.

[CR53] Thaler JS, Humphrey PT, Whiteman NK (2012). Evolution of jasmonate and salicylate signal crosstalk. Trends Plant Sci.

[CR54] Throop HL, Lerdau MT (2004). Effects of nitrogen deposition on insect herbivory: Implications for community and ecosystem processes. Ecosystems.

[CR55] Valifard M, Mohsenzadeh S, Niazi A, Moghadam A (2015). Phenylalanine ammonia lyase isolation and functional analysis of phenylpropanoid pathway under salinity stress in *Salvia* species. Aust J Crop Sci.

[CR56] Warabieda W (2015). The effect of methyl jasmonate and acibenzolar-S-methyl on the populations of the European red mite (*Panonychus ulmi* Koch) and *Typhlodromus pyri* Scheut. in apple orchards, as well as on the yield and growth of apple trees. Int J Acarol.

[CR57] Warabieda W, Olszak RW (2010). Effect of exogenous methyl jasmonate on numerical growth of the population of the two-spotted spider mite (*Tetranychus urticae* Koch) on strawberry plants and young apple trees. J Plant Prot Res.

[CR58] Warabieda W, Markiewicz M, Wójcik D, Pulawska J (2015). Mutual relations between jasmonic acid and Acibenzolar-S-methyl in the induction of resistance to fire blight in apple trees. J Plant Pathol.

[CR59] Wasternack C, Hause B (2013). Jasmonates: biosynthesis, perception, signal transduction and action in plant stress response, growth and development. An update to the 2007 review in *Annals of Botany*. Ann Bot.

[CR60] Wermelinger B, Oertli JJ, Delucchi V (1985). The effects of host plant N fertilization on the biology of the two-spotted spider mite, *Tetranychus urticae*. Entomol Exp Appl.

[CR61] Wójcik P (2018). Response of ‘Red Delicious’ apple trees to different liming strategies after drip fertigation with ammonium nitrate. J Elem.

[CR62] Xu T, Wang J, Luo S (2005). Cloning of the key genes in maize oxylipins pathways and their roles in herbivore induced defense. Chin Sci Bull.

[CR63] Yang XY, Jiang WJ, Yu HJ (2012). The expression profiling of the Lipoxygenase (LOX) family genes during fruit development, abiotic stress and hormonal treatments in cucumber (*Cucumis sativus* L.). Int J Mol Sci.

[CR64] Zhang Y, Shi X, Li B, Zhang Q, Liang W, Wang C (2016). Salicylic acid confers enhanced resistance to Glomerella leaf spot in apple. Plant Physiol Biochem.

[CR65] Zhurov V, Navarro M, Bruinsma KA, Arbona V, Santamaria ME (2014). Reciprocal responses in the interaction between Arabidopsis and the cell-content-feeding chelicerate herbivore spider mite. Plant Physiol.

